# Diet high in branched-chain amino acid promotes PDAC development by USP1-mediated BCAT2 stabilization

**DOI:** 10.1093/nsr/nwab212

**Published:** 2021-11-26

**Authors:** Jin-Tao Li, Kai-Yue Li, Ying Su, Yuan Shen, Ming-Zhu Lei, Fan Zhang, Miao Yin, Zheng-Jun Chen, Wen-Yu Wen, Wei-Guo Hu, Dan Su, Jia Qu, Qun-Ying Lei

**Affiliations:** Fudan University Shanghai Cancer Center and Institutes of Biomedical Sciences; Cancer Institutes; Key Laboratory of Breast Cancer in Shanghai; Shanghai Key Laboratory of Medical Epigenetics; International Co-Laboratory of Medical Epigenetics and Metabolism, Ministry of Science and Technology, Shanghai Medical College, Fudan University, Shanghai 200032, China; Fudan University Shanghai Cancer Center and Institutes of Biomedical Sciences; Cancer Institutes; Key Laboratory of Breast Cancer in Shanghai; Shanghai Key Laboratory of Medical Epigenetics; International Co-Laboratory of Medical Epigenetics and Metabolism, Ministry of Science and Technology, Shanghai Medical College, Fudan University, Shanghai 200032, China; Fudan University Shanghai Cancer Center and Institutes of Biomedical Sciences; Cancer Institutes; Key Laboratory of Breast Cancer in Shanghai; Shanghai Key Laboratory of Medical Epigenetics; International Co-Laboratory of Medical Epigenetics and Metabolism, Ministry of Science and Technology, Shanghai Medical College, Fudan University, Shanghai 200032, China; Fudan University Shanghai Cancer Center and Institutes of Biomedical Sciences; Cancer Institutes; Key Laboratory of Breast Cancer in Shanghai; Shanghai Key Laboratory of Medical Epigenetics; International Co-Laboratory of Medical Epigenetics and Metabolism, Ministry of Science and Technology, Shanghai Medical College, Fudan University, Shanghai 200032, China; Fudan University Shanghai Cancer Center and Institutes of Biomedical Sciences; Cancer Institutes; Key Laboratory of Breast Cancer in Shanghai; Shanghai Key Laboratory of Medical Epigenetics; International Co-Laboratory of Medical Epigenetics and Metabolism, Ministry of Science and Technology, Shanghai Medical College, Fudan University, Shanghai 200032, China; Fudan University Shanghai Cancer Center and Institutes of Biomedical Sciences; Cancer Institutes; Key Laboratory of Breast Cancer in Shanghai; Shanghai Key Laboratory of Medical Epigenetics; International Co-Laboratory of Medical Epigenetics and Metabolism, Ministry of Science and Technology, Shanghai Medical College, Fudan University, Shanghai 200032, China; Fudan University Shanghai Cancer Center and Institutes of Biomedical Sciences; Cancer Institutes; Key Laboratory of Breast Cancer in Shanghai; Shanghai Key Laboratory of Medical Epigenetics; International Co-Laboratory of Medical Epigenetics and Metabolism, Ministry of Science and Technology, Shanghai Medical College, Fudan University, Shanghai 200032, China; State Key Laboratory of Cell Biology, Shanghai Institute of Biochemistry and Cell Biology, Center for Excellence in Molecular Cell Science, Chinese Academy of Sciences, Shanghai 200031, China; Fudan University Shanghai Cancer Center and Institutes of Biomedical Sciences; Cancer Institutes; Key Laboratory of Breast Cancer in Shanghai; Shanghai Key Laboratory of Medical Epigenetics; International Co-Laboratory of Medical Epigenetics and Metabolism, Ministry of Science and Technology, Shanghai Medical College, Fudan University, Shanghai 200032, China; Fudan University Shanghai Cancer Center and Institutes of Biomedical Sciences; Cancer Institutes; Key Laboratory of Breast Cancer in Shanghai; Shanghai Key Laboratory of Medical Epigenetics; International Co-Laboratory of Medical Epigenetics and Metabolism, Ministry of Science and Technology, Shanghai Medical College, Fudan University, Shanghai 200032, China; Cancer Research Institute, Zhejiang Cancer Hospital and Key Laboratory Diagnosis and Treatment Technology on Thoracic Oncology of Zhejiang Province, Hangzhou 310022, China; Fudan University Shanghai Cancer Center and Institutes of Biomedical Sciences; Cancer Institutes; Key Laboratory of Breast Cancer in Shanghai; Shanghai Key Laboratory of Medical Epigenetics; International Co-Laboratory of Medical Epigenetics and Metabolism, Ministry of Science and Technology, Shanghai Medical College, Fudan University, Shanghai 200032, China; Fudan University Shanghai Cancer Center and Institutes of Biomedical Sciences; Cancer Institutes; Key Laboratory of Breast Cancer in Shanghai; Shanghai Key Laboratory of Medical Epigenetics; International Co-Laboratory of Medical Epigenetics and Metabolism, Ministry of Science and Technology, Shanghai Medical College, Fudan University, Shanghai 200032, China; Department of Oncology, Shanghai Medical College, Fudan University, Shanghai 200032, China; State Key Laboratory of Medical Neurobiology, Fudan University, Shanghai 200032, China

**Keywords:** USP1, deubiquitylation, BCAT2, PanIN, PDAC

## Abstract

BCAT2-mediated branched-chain amino acid (BCAA) catabolism is critical for pancreatic ductal adenocarcinoma (PDAC) development, especially at an early stage. However, whether a high-BCAA diet promotes PDAC development *in vivo*, and the underlying mechanism of BCAT2 upregulation, remain undefined. Here, we find that a high-BCAA diet promotes pancreatic intraepithelial neoplasia (PanIN) progression in *LSL-Kras^G12D/+^*; *Pdx1-Cre* (KC) mice. Moreover, we screened with an available deubiquitylase library which contains 31 members of USP family and identified that USP1 deubiquitylates BCAT2 at the K229 site. Furthermore, BCAA increases USP1 protein at the translational level via the GCN2-eIF2α pathway both *in vitro* and *in vivo*. More importantly, USP1 inhibition recedes cell proliferation and clone formation in PDAC cells and attenuates pancreas tumor growth in an orthotopic transplanted mice model. Consistently, a positive correlation between USP1 and BCAT2 is found in KC; *LSL-Kras^G12D/+^*; *p53^flox/+^*; *Pdx1-Cre* mice and clinical samples. Thus, a therapeutic targeting USP1-BCAT2-BCAA metabolic axis could be considered as a rational strategy for treatment of PDAC and precisive dietary intervention of BCAA has potentially translational significance.

## INTRODUCTION

Cancer cells obtain the capability to rewire a metabolic pathway, producing energy and indispensable biomass to sustain uncontrolled proliferation of cancer cells upon various stressful stimuli. One century after the disclosure of enhanced glycolysis in cancer cells, the substantial contribution of plentiful nutrients/metabolites, including amino acids, lipids and acetate as well as others, to cancer development has been demonstrated [1,2]. The breakdown of amino acids has been recognized as a carbon and nitrogen source that is essential for energy supplement, redox protection and cellular component synthesis in cancer cells. In pancreatic ductal adenocarcinoma (PDAC), the metabolism of Ala, Pro, Gln, Asp and Ser, as well as BCAT2-mediated branched-chain amino acid (BCAA), has been usurped for malignant progression [3–11]. Therefore, effective therapy of PDAC would benefit from pharmaceutical and/or dietary intervention with regard to irregular metabolism of amino acids [4,10,11].

The conversion of BCAAs (isoleucine, leucine and valine) to branched-chain α-keto acids (BCKAs) is catalyzed by BCAA aminotransferase (BCAT). In mammal cells, two major isoforms of BCAT, BCAT1 or 2, are present in cytoplasm or mitochondrion, respectively, directing amino group transference [12–14]. The involvement of BCAA metabolism remodeling is associated with metabolic disorders, including different cancer types [15–20]. For example, upregulation of plasma BCAA has been suggested as a predictor for PDAC occurrence [21]. In stroma-rich pancreatic cancers, elevated BCAT1 protein levels in cancer-associated fibroblast (CAF) cells led to an increase in secreted BCKA, fueling PDAC cell growth by maintenance of downstream metabolite pools [3]. Meanwhile, other reports showed that overexpressed BCAT2 in PDAC cells enhanced cell proliferation and survival *in vitro* and *in vivo* [3,4,7]. On the other hand, the major source of nutrients/metabolites in the body comes from diet. Diet intervention for treatment of cancer is emerging. It remains unknown whether a diet with high BCAA content promotes PDAC progression *in vivo*.

Mechanistically, our recent work revealed that K-Ras (KRAS) mutation disrupted BCAT2 ubiquitylation, leading to its stabilization in PDAC cells. But the deubiquitylase (DUB) of BCAT2 has not been identified yet. It is notable that K229 is an active site of BCAT2 that binds with its cofactor pyridoxal phosphate (PLP) [22]. Ubiquitin specific peptidase 1 (USP1), a sub-type of DUB, displays diverse cellular functions and is essential for cellular homoeostasis and the response to DNA damage [23,24]. USP1 regulates the cell cycle via reducing the degradation of phosphorylated checkpoint kinase 1 (CHK1) and maintaining its activity [25]. Removal of polyubiquitylation of Nucleotide-Binding Oligomerization Domain-like receptor protein 3 (NLRP3) by USP1 enhances cellular NLRP3 levels, which is indispensable for inflammasome assembly and activation [26]. In addition, USP1 is upregulated in multiple cancers, like osteosarcoma, breast cancer, hepatic carcinoma and colorectal cancer [27–30]. Overall, USP1 might be a promising therapeutic target in cancers. However, few studies have examined the mechanism and function of USP1 in PDAC. In eukaryotic cells, the mammalian target of rapamycin (mTOR) and general control nonderepressible 2 (GCN2) are two conserved signaling pathways that sense and respond to the fluctuation of amino acids, especially BCAA [31]. GCN2 senses and is activated by uncharged tRNA in amino-acid-limited conditions. Activated GCN2 phosphorylates eukaryotic translation initiation factor 2 subunit α (eIF2α) to block general protein synthesis and subsequently activate activating transcription factor 4 (ATF4). ATF4 drives the transcription of genes involved in autophagy, amino acid biosynthesis and transport to adapt to amino acid limitation. Here, we found that the BCAA signal enhances the USP1 protein level at translational level via the GCN2- eIF2α pathway *in vitro* and *in vivo*. Our work revealed that a high-BCAA-content diet increases the USP1 protein level and subsequently stabilizes BCAT2 to enhance BCAA utilization, thus leading to PDAC progression.

## RESULTS

### Diet with high BCAA content promotes PanIN progression

**Figure 1.**
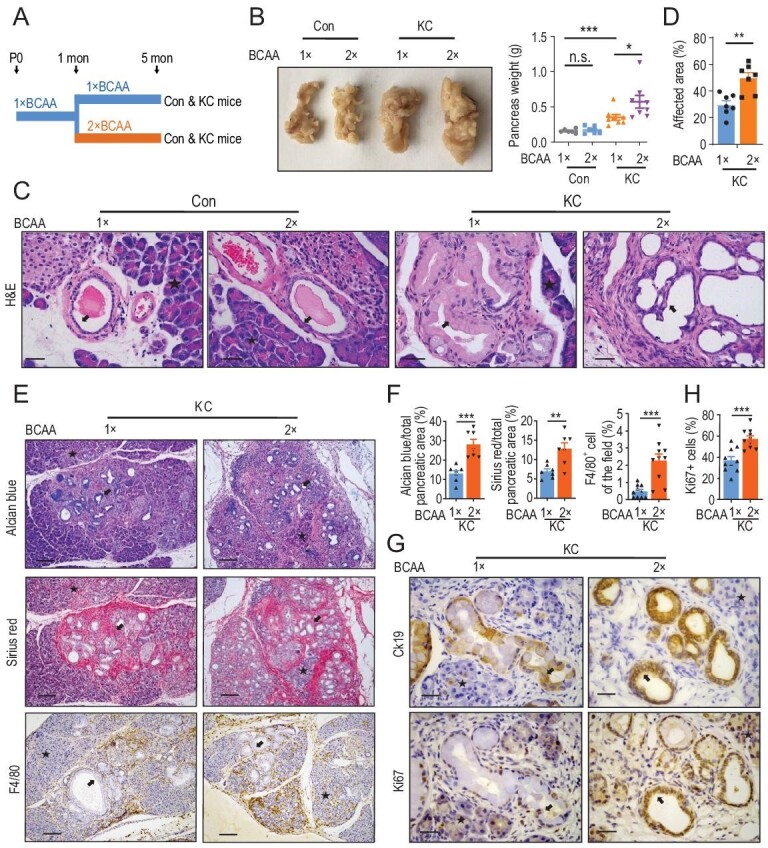
A diet with high BCAA content promotes PanIN progression. (A) Schematic overview of the feeding of a high-BCAA diet in a mice model. (B) A high-BCAA diet increases the pancreatic weight of KC mice, shown by a pancreas image and distributions of mouse pancreas weight (mean ± standard error of mean (SEM) of *n* = 6–8 biologically independent animals, one-way ANOVA test). (C and D) A high-BCAA diet promotes PanIN progression. (C) Representative H&E staining images and (D) quantification of the affected area of the pancreas (mean ± SEM of *n* = 7 biologically independent fields, two-tailed t-test). (E and H) Representative images of and quantification of (E and F) Alcian blue, Sirius red and F4/80, and (G and H) Ki67 (mean ± SEM of *n* = 7–10 biologically independent fields, two-tailed t-test). In (C and G) scale bars, 12.5 μm; in (E) scale bars, 50 μm. n.s. donates for no significance, ^*^P < 0.05, ^**^P < 0.01, ^***^P < 0.001 and ^****^P < 0.0001.

Our recent work found that a BCAA-restricted diet significantly inhibits PDAC development [4]. Next, we wonder whether a diet with high BCAA content would promote PanIN lesions. To this end, we used a bespoke diet containing 2-fold content of BCAA compared to normal diet. Mice were administered this diet at the age of 1 month and sacrificed at the age of 5 months (Fig. 1A). A diet with high BCAA content increased the BCAA concentration in both the plasma and pancreas of control and KC mice (Fig. S1A and B). Interestingly, the BCAA concentration in the pancreas was higher in KC mice compared to control mice with a normal BCAA diet (Fig. S1B). The pancreas weight of KC mice fed with a high-BCAA diet was significantly increased, while a high-BCAA diet had no effect on pancreas weight in the control group (Fig. 1B). Body weight was not affected in the control and KC groups fed with a high-BCAA diet (Fig. S1C). In addition, a high-BCAA diet had no effect on the glucose tolerance test (GTT) or insulin tolerance test (ITT) in both control and KC mice (Fig. S1D and E). No significant histological changes were observed in the heart, liver, spleen, lung, kidney or muscle of mice treated with a high-BCAA diet (Fig. S1F). Notably, a high-BCAA diet increased the overall burden of PanIN lesions in KC mice (Fig. 1C and D). In particular, histological examination demonstrated that PanIN lesions in one in eight KC mice treated with a high-BCAA diet developed into PDAC (Fig. 1C). As the precursor lesions of PDAC, PanIN is characterized by abundant mucin in ductal cells, extracellular matrix (ECM) deposition and macrophage infiltration, which can be detected by staining of Alcian blue, Sirius red and F4/80, respectively [32]. Evaluation of the staining intensity of Alcian blue, Sirius red and F4/80 revealed that the high-BCAA diet significantly promoted advancement of PanIN lesions in KC mice (Fig. 1E and F). Furthermore, immunohistochemistry (IHC) analyses of cytokeratin19 (CK19) and Ki67 expression, presenting as a pancreatic ductal epithelial cell marker and cell proliferation marker, respectively, in serial sections, showed that a high-BCAA diet significantly boosted proliferation of ductal cells within KRAS^G12D^-driven PanIN lesions (Fig. 1G and H). Altogether, we found that a high-BCAA diet substantially fostered PanIN progression in KC mice.

### USP1 is the DUB of BCAT2 in response to BCAA availability

**Figure 2.**
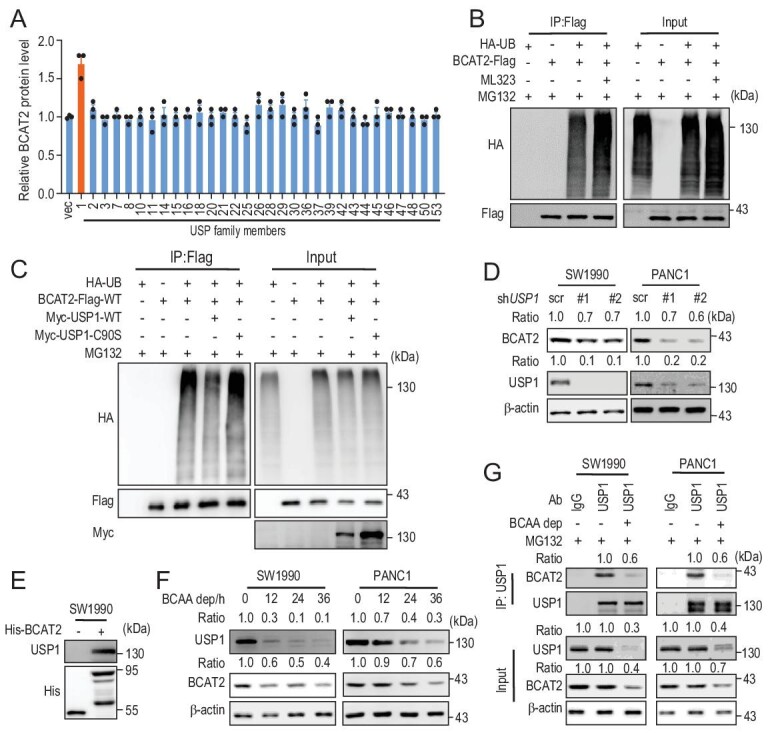
USP1 is the DUB of BCAT2 in response to BCAA availability. (A) Screening assay with DUB library revealed upregulation of the BCAT2 protein level by USP1. Mean ± SEM of *n* = 3 biologically independent experiments. (B) ML323 (USP1 specific inhibitor, 5 μm, 24 h) increases BCAT2 ubiquitylation. (C) USP1^WT^ but not USP1^C90S^ mutant decreases BCAT2 ubiquitylation. (D) *USP1* knockdown decreases the BCAT2 protein level in SW1990 cells and PANC1 cells. ‘Ratio’, upper panel: BCAT2/β-actin; lower panel: USP1/β-actin. (E) His-BCAT2 pulls down USP1 in SW1990 cells. (F) BCAA deprivation decreases USP1 and BCAT2 protein levels. ‘Ratio’, upper panel: USP1/β-actin; lower panel: BCAT2/β-actin. (G) BCAA deprivation disrupts the interaction between USP1 and BCAT2. ‘Ratio’, upper panel: BCAT2/USP1; middle panel: USP1/β-actin; lower panel: BCAT2/β-actin. Data in (B–G) are representative of three biologically independent experiments.

Protein ubiquitylation is a reversible reaction. Our recent work demonstrated ubiquitylation of BCAT2 in PDAC cells [4]. However, the DUB of BCAT2 remains unknown. We performed screening identification with the available DUB library and found that ectopic USP1 expression increased the BCAT2 protein level under our tested condition (Fig. 2A and Fig. S2A), indicating that USP1 was a potential DUB of BCAT2. Furthermore, we found that USP1 increased the BCAT2 protein level in a dose-dependent manner (Fig. S2B). Moreover, we isolated mitochondrial and found that USP1 co-localized with BCAT2 in mitochondria (Fig. S2C). Intriguingly, both BCAT2 and USP1 protein levels were consistently upregulated in PDAC cells compared to H6C7 cells and HPNE cells, which are immortalized normal pancreas ductal cells (Fig. S2D). Next, we found that treatment with ML323, a specific inhibitor of USP1 [33], enhanced BCAT2 ubiquitylation (Fig. 2B) whereas USP1 wild-type but not enzyme-dead C90S mutant overexpression decreased BCAT2 ubiquitylation (Fig. 2C). Conversely, *USP1* knockdown decreased the BCAT2 protein level in SW1990 cells and PANC1 cells (Fig. 2D). We further confirmed that USP1 interacted with prokaryotic purified BCAT2 *in vitro* (Fig. 2E). Notably, the USP1 protein level was downregulated by BCAA deprivation in a time-dependent manner in SW1990 cells and PANC1 cells (Fig. 2F). Meanwhile, treatment with high-concentration BCAA upregulated USP1 and BCAT2 protein levels in a dose-dependent manner (Fig. S2E). However, the BCAT1 protein level was not affected in response to BCAA under our tested conditions (Fig. S2F). Furthermore, BCAA deprivation interrupted the interaction between BCAT2 and USP1 endogenously (Fig. 2G). Taken together, our results demonstrate that USP1 is a DUB of BCAT2 in response to BCAA availability.

### USP1 deubiquitylates BCAT2 at the K229 site

**Figure 3.**
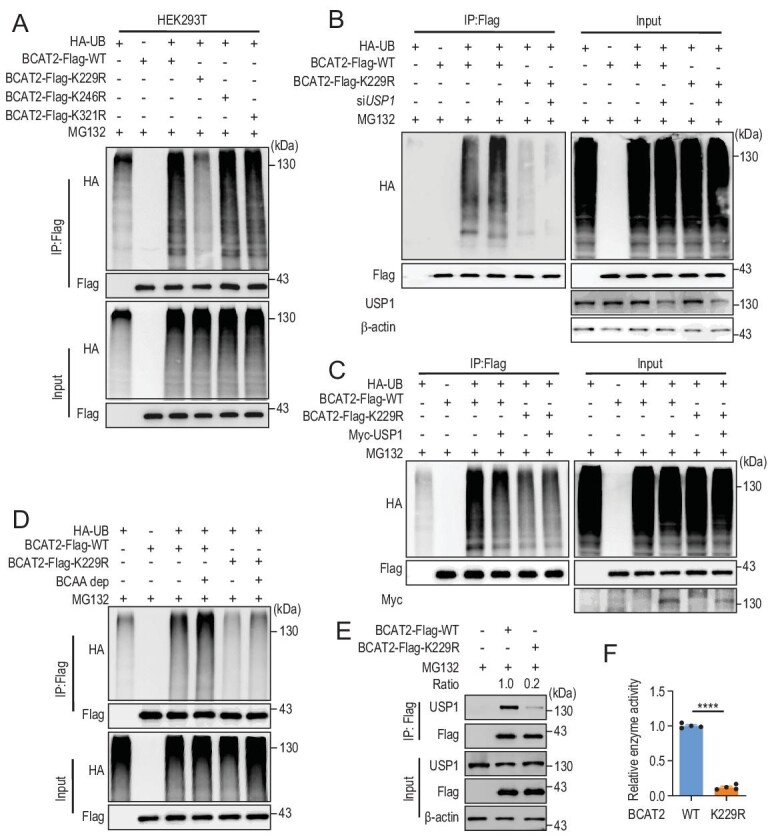
USP1 deubiquitylates BCAT2 at the K229 site. (A) K229R mutation decreases the BCAT2 ubiquitylation level in HEK293T cells. (B) *USP1* knockdown enhances ubiquitylation of BCAT2^WT^ but not BCAT2^K229R^ mutant. (C) USP1 decreases ubiquitylation of BCAT2^WT^ but not BCAT2^K229R^. (D) BCAA deprivation enhances ubiquitylation of BCAT2^WT^ but not BCAT2^K229R^ mutant. (E) K229R mutant disrupts the semi-endogenous binding between BCAT2 and USP1. ‘Ratio’, USP1/Flag. (F) K229R mutant significantly attenuates BCAT2 enzyme activity. Mean ± SEM of *n* = 4 biologically independent experiments, two-tailed t-test. Data in (A–E) are representative of three biologically independent experiments. ^****^P < 0.0001.

PhosphoSitePlus (https://www.phosphosite.org/) analysis predicted that K229 (preprotein site K229, mature site K202), K246 (preprotein site K246, mature site K219) and K321 (preprotein site K321, mature site K294) are potential ubiquitylation sites of BCAT2 [34]. Therefore, these three sites were mutated individually. Only the K229R mutation caused significant attenuation of BCAT2 ubiquitylation (Fig. 3A). Furthermore, *USP1* knockdown enhanced ubiquitylation of the BCAT2^WT^ but not BCAT2^K229R^ mutant (Fig. 3B). Consistently, ML323 treatment enhanced ubiquitylation of the BCAT2^WT^ but not BCAT2^K229R^ mutant (Fig. S3A). In contrast, ectopic USP1 expression decreased ubiquitylation of the BCAT2^WT^ but not BCAT2^K229R^ mutant (Fig. 3C). In addition, the effect of BCAA on BCAT2 ubiquitylation was measured. Ubiquitylation of the BCAT2^WT^ but not BCAT2^K229R^ mutant was dramatically elevated upon BCAA deprivation (Fig. 3D). Meanwhile, ectopic K229R mutant expression prolonged BCAT2 protein half-life in HEK293T cells (Fig. S3B and C) and disrupted the binding between BCAT2 and USP1 (Fig. 3E). As mentioned before, K229 is an active site of BCAT2. We examined the effect of mutation at K229 on its catalytic capability and found that the K229R mutant dramatically attenuated BCAT2 enzyme activity (Fig. 3F). Taken together, these results suggest that K229 is a major ubiquitylation site of BCAT2 under our tested conditions.

### BCAA upregulates USP1 via GCN2-eIF2**α** at the translational level

**Figure 4.**
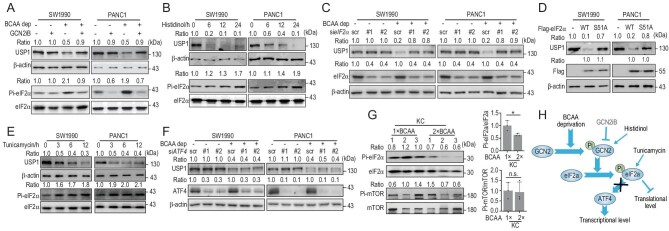
BCAA increases USP1 protein via the GCN2-eIF2α pathway. (A) GCN2 inhibitor (GCN2iB, 5 μM) rescues the USP1 protein level with BCAA deprivation. ‘Ratio’, upper panel: USP1/β-actin; lower panel: Pi-eIF2α/eIF2α. (B) Histidinol (2 mM) decreases the USP1 protein level in a time-dependent manner. ‘Ratio’, upper panel: USP1/β-actin; lower panel: Pi-eIF2α/eIF2α. (C) Knockdown of *eIF2*α rescues the USP1 protein level under the BCAA deprivation condition. ‘Ratio’, upper panel: USP1/β-actin; lower panel: eIF2α/β-actin. (D) Overexpression of eIF2α wild-type but not enzyme-dead S51A mutant dramatically decreases the USP1 protein level. ‘Ratio’, upper panel: USP1/β-actin; lower panel: Flag/β-actin. (E) Tunicamycin (5 μg/mL) attenuates the USP1 protein level in a time-dependent manner. ‘Ratio’, upper panel: USP1/β-actin; lower panel: Pi-eIF2α/eIF2α. (F) *ATF4* knockdown fails to rescue the USP1 protein level under the BCAA deprivation condition. ‘Ratio’, upper panel: USP1/β-actin; lower panel: ATF4/β-actin. (G) A higher BCAA diet decreases phosphorylation of eIF2α and has no effect on mTOR in KC mice. Mean ± SEM of *n* = 3 biologically independent experiments, two-tailed t-test. (H) Working model. BCAA deprivation activates the GCN2-eIF2α pathway and blocks USP1 protein synthesis. Data in (A–G) are representative of three biologically independent experiments. n.s. donates for no significance, ^*^P < 0.05.

Next, we explored the underlying mechanism of how BCAA regulates USP1. We found that neither the *BCAT2* nor *USP1* mRNA level was affected under BCAA deprivation at 12, 24 and 36 hours (h) in SW1990 cells (Fig. S4A), and at 12 and 24 h in PANC1 cells (Fig. S4A), suggesting that the USP1 protein level may be regulated at the translational level or post-translational modification (PTM) level. However, we found that inhibitors of lysosome and proteasome pathways failed to rescue the USP1 protein level under the BCAA deprivation condition, indicating that USP1 expression is not regulated via PTM (Fig. S4B). It is well known that mTOR and the GCN2-eIF2α pathway respond to BCAA signaling. Therefore, we employed rapamycin to treat PANC1 cells, and found that rapamycin treatment had no effect on the USP1 protein level under our tested conditions (Fig. S4C). Interestingly, GCN2iB (a specific inhibitor of GCN2) treatment dramatically rescued the USP1 protein level under BCAA deprivation (Fig. 4A), suggesting that the BCAA signal may regulate USP1 via the GCN2-eIF2α pathway. Next, we employed histidinol, which is a structural analogue of histidine that inhibits the charging of hitidyl-tRNA and results in activation of GCN2 in the absence of histidine. We found that histidinol treatment dramatically attenuated the USP1 protein level in a time-dependent manner in SW1990 cells and PANC1 cells (Fig. 4B). Moreover, *eIF2α* knockdown rescued the USP1 protein level upon BCAA deprivation (Fig. 4C). Conversely, overexpression of the eIF2α wild-type but not enzyme-dead S51A mutant dramatically decreased the USP1 protein level (Fig. 4D). Tunicamycin, an activator of PRKR-like endoplasmic reticulum kinase, which is another kinase of eIF2α, can subsequently activate eIF2α. We found that tunicamycin treatment attenuated the USP1 protein level in a time-dependent manner (Fig. 4E). Furthermore, *ATF4* knockdown failed to rescue the USP1 protein level under the BCAA deprivation condition (Fig. 4F), suggesting that the ATF4 downstream pathway is not involved in USP1 protein synthesis. Consistently, the phosphorylation level of eIF2α was downregulated in KC mice fed with a 2-fold BCAA diet, whereas the mTOR phosphorylation level was not affected in KC mice with a 2-fold BCAA diet (Fig. 4G). Meanwhile, neither the *Bcat2* nor *Usp1* mRNA level was affected in KC mice treated with a 2-fold BCAA diet (Fig. S4D). Altogether, our results demonstrate that BCAA upregulates USP1 protein synthesis via the GCN2-eIF2α pathway at the translational level both *in vitro* and *in vivo* (Fig. 4H).

### USP1 promotes PDAC cell proliferation via BCAA catabolism

**Figure 5.**
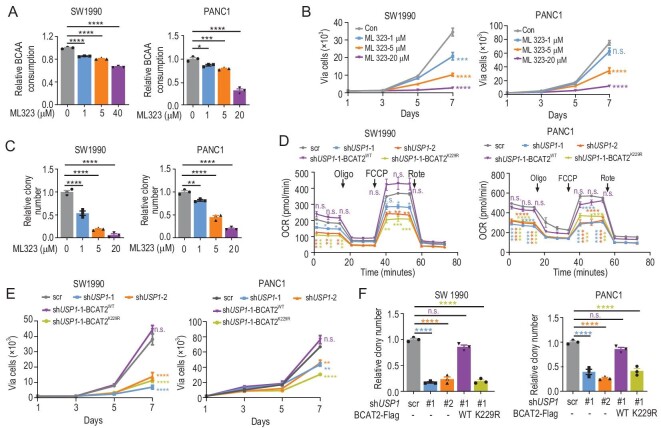
USP1 promotes PDAC cell proliferation via BCAA catabolism. (A) ML323 decreases BCAA consumption in SW1990 cells and PANC1 cells in a dose-dependent manner. Mean ± SEM of *n* = 3 biologically independent experiments, one-way ANOVA test. (B) ML323 attenuates cell proliferation in SW1990 cells and PANC1 cells in a dose-dependent manner. Mean ± SEM of *n* = 4 biologically independent experiments, one-way ANOVA test. (C) ML323 attenuates colony formation in SW1990 cells and PANC1 cells in a dose-dependent manner. Mean ± SEM of *n* = 3 biologically independent experiments, one-way ANOVA test. (D) BCAT2^WT^ but not BCAT2^K229R^ putback rescues OCR in *USP1* knockdown cells. Mean ± SEM of *n* = 3 biologically independent experiments, one-way ANOVA test. (E and F) BCAT2^WT^ but not BCAT2^K229R^ putback recovered *USP1* knockdown-induced reduction of (E) cell proliferation and (F) clone formation in SW1990 and PANC1 cells. Mean ± SEM of *n* = 4 (E), *n* = 3 (F) biologically independent experiments, one-way ANOVA test. n.s. donates for no significance, ^*^P < 0.05, ^**^P < 0.01, ^***^P < 0.001 and ^****^P < 0.0001.

Next, we explored the effect of USP1 on PDAC cell growth. ML323 treatment impeded BCAA consumption in a dose-dependent manner in SW1990 cells and PANC1 cells (Fig. 5A). BCAA deprivation decreased cell proliferation in SW1990 cells and PANC1 cells (Fig. S5A). USP1 overexpression enhanced cell proliferation in PDAC cells (Fig. S5B and C). Next, we performed Ki-67 staining in SW1990 cells to exclude toxicity of ML323 and found that ML323 treatment attenuated cell proliferation in a dose-dependent manner (Fig. S5D and E). Simultaneously, proliferation (Fig. 5B) and clone formation (Fig. 5C and Fig. S5F) of SW1990 cells and PANC1 cells were suppressed by ML323 in a dose-dependent manner as well. To uncover effects of USP1 on metabolic flux, we determined the extracellular acidification rate (ECAR) and oxygen consumption rate (OCR), which are indicators of glycolysis flux and mitochondrial respiration, respectively. *USP1* knockdown did not affect ECAR but it decreased OCR in PDAC cells (Fig. 5D and Fig. S5G and H). As expected, BCAT2^WT^ but not BCAT2^K229R^ putback rescued OCR in SW1990 cells and PANC1 cells (Fig. 5D and Fig. S5G). Consistently, BCAT2^WT^ but not BCAT2^K229R^ putback revived cell proliferation (Fig. 5E) and clone formation (Fig. 5F and Fig. S5I) in cells with *USP1* silencing. Taken together, USP1 promotes PDAC cell proliferation via aiding BCAA catabolism.

### Positive correlation between the expression of USP1 and BCAT2 in PDAC

**Figure 6.**
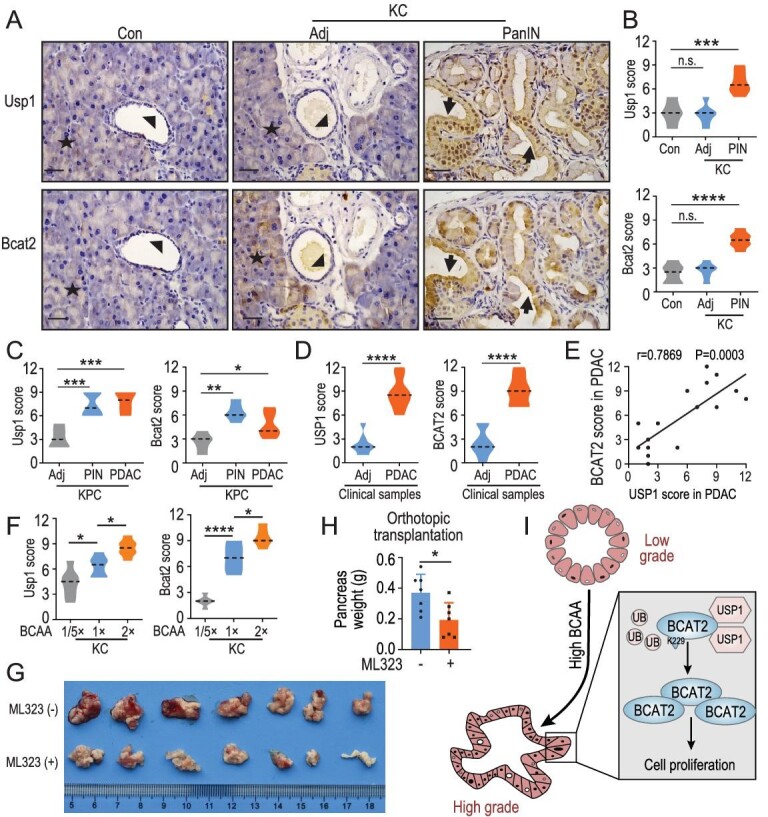
Positive correlation between expression of USP1 and BCAT2 in PDAC. (A) Representative images and (B) quantification of Usp1- and Bcat2-staining of serial sections in KC mice. Acinar, adjacent duct and PDAC area are indicated as asterisk, arrowhead and arrow, respectively. (C) Quantification of Usp1- and Bcat2-staining of serial sections in KPC mice. (D) Quantification of USP1- and BCAT2-staining of serial sections from adjacent tissues and PDAC specimens. (E) Positive correlation between USP1 and BCAT2 protein levels in PDAC samples (*n* = 10 biologically independent patients; Spearman correlation, two-tailed). (F) Quantification of Usp1- and Bcat2-staining of serial sections in KC mice treated with the indicated BCAA diet. (G) Photo and (H) quantification of orthotopical transplantation in C57 mice treated with ML323. Mean ± SEM of *n* = 7 (H) biologically independent samples, two tailed t-test. (I) Working model. High BCAA increases the USP1 protein level and attenuates ubiquitylation of BCAT2 at the K229 site, stabilizing BCAT2 to promote PDAC progression. Mean ± SEM of *n* = 6 (B), *n* = 5 (C), *n* = 10 (consistently, the phosphorylation level of eIF2α was D) and *n* = 6 (F) biologically independent samples, median indicated as dotted lines, one-way ANOVA test. Scale bar: 12.5 μm. n.s. donates for no significance, ^*^P < 0.05, ^**^P < 0.01, ^***^P < 0.001 and ^****^P < 0.0001.

To determine the relationship between the protein expression levels of USP1 and BCAT2 in PDAC tissue samples, we performed IHC staining and demonstrated that Usp1 and Bcat2 protein levels were detectable in pancreatic acinar cells but not in normal ductal cells in a control group (Fig. 6A). Of note, significantly increased protein levels of Usp1 and Bcat2 were observed in the PanIN ductal cells of KC mice (Fig. 6A and B). Also, Usp1 and Bcat2 protein levels were upregulated in ductal cells within PanIN lesions and PDAC areas in LSL-Kras G12D/+; p53 flox/+; Pdx1-Cre (KPC) mice (Fig. 6C and Fig. S6A). Moreover, we employed 10 clinical samples from human PDAC, and found that USP1 and BCAT2 protein levels were ubiquitously elevated in PDAC ductal cells while the signal intensities of USP1 and BCAT2 staining were undetectable in adjacent normal ductal cells (Fig. 6D and Fig. S6B), and a positive correlation was revealed between the expression levels of BCAT2 and USP1 protein in PDAC samples (Fig. 6E). Further data set analyses with the cancer genome atlas (TCGA) demonstrated that a high USP1 expression level predicts poor overall survival of PDAC patients (Fig. S6C). However, the BCAT2 expression level was not correlated with patient survival (Fig. S6C). This may be due to availability of genes, but not protein expression level from TCGA. Conclusively, USP1 and BCAT2 protein levels are upregulated in PanIN and PDAC ductal cells and display positive correlation.

Our previous data demonstrated that a diet with reduced BCAA blocks PanIN progression in KC mice [4]. We thus asked whether dietary intervention affects Usp1 and Bcat2 protein levels *in vivo*. Indeed, a low-BCAA diet decreased the Usp1 protein level in PDAC generated by orthotopic transplantation, concomitantly with cancer cell proliferation arresting (Fig. S6D and E). Consistently, we found that a low-BCAA diet fundamentally decreased both Usp1 and Bcat2 protein levels in ductal cells within PanIN lesions in KC mice (Fig. 6F and Fig. S6F). Conversely, a high-BCAA diet led to upregulation of Usp1 and Bcat2 protein levels in ductal cells within PanIN lesions of KC mice (Fig. 6F and Fig. S6F). Furthermore, IHC staining of Bcat1 revealed that Bcat1 was not detectable in acinar cells, islet cells, adjacent normal ductal cells or PanIN ductal cells in KC mice (Fig. S6G and H). A higher BCAA diet had no effect on Bcat1 expression in PanIN ductal cells in KC mice (Fig. S6G and H). Meanwhile, a higher BCAA diet enhanced the TGF-β protein level, but had no effect on Bcat1 and BCAA transporter Slc7a5 in KC mice fed with a 2-fold BCAA diet (Fig. S6I and J). Moreover, ML323 dramatically attenuated tumor weight in an orthotopically transplanted mice model (Fig. 6G and H). Dietary BCAA is critical for supporting Usp1 and Bcat2 protein levels *in vivo*, and targeting the USP1-BCAT2-BCAA catabolic axis could be a rational strategy for PDAC therapy.

## DISCUSSION

BCAAs, including leucine, isoleucine and valine, are essential amino acids provided by food or supplementation. In health, BCAA supplementation contributes to the maintenance of tissue homeostasis, like muscle building, thermogenesis in brown adipose tissue and exercise recovery [35]. With the depiction of the involvement of BCAA in various metabolic disorders like obesity, insulin resistance and, particularly, cancer development, dietary intervention of BCAA intake is considered as a potentially therapeutic strategy against cancer as well as other metabolic diseases. BCAA supplementation has been widely used for muscle strengthening. Muscle loss, the common condition of cachexia occurring in advanced cancers, requires sufficient intake of protein and amino acids to be ameliorated. However, it is reported that genetic disorders, diabetes and dietary factors, including alcohol abuse and high fat/protein intake, increase the risk of PDAC [36]. Meanwhile, an elevated BCAA concentration was found in the early stage of PDAC in human patients [21]. The accumulating knowledge of the effect of BCAA on cancer is a reminder to carefully evaluate the application of BCAA supplementation in treatment. One study showed that BCAA supplementation lessens fibrosis and inhibits liver cancer progression and recurrence in mice [37–39]. However, other studies reported that dietary BCAA levels seem to have a positive correlation with the development of liver cancer and colorectal cancer [40,41]. These contradictory results indicate that a dietary BCAA regimen might be stage- and/or tissue-dependent. In PDAC, the concentration of plasma BCAA was elevated at the early stage before diagnosis, and BCAT2 was upregulated in PanIN ductal cells. However, targeted drugs for BCAT2 are still lacking, the tracing of the diets of pancreatic cancer patients at an early stage still needs further study, and direct evidence to prove that dietary BCAA promotes PDAC progression *in vivo* needs to be addressed. Our findings demonstrate that a long-term intake of high BCAA promotes PanIN progression and even leads to tumorigenesis in a mice model harboring KRAS mutation (Fig. 6I). Our results suggest that dietary intervention of BCAA could be applied at a very early stage, i.e. PanIN lesions, to effectively prevent PDAC development.

BCAT includes two isozymes, BCAT1 and BCAT2, and functions as the initial catabolism enzyme of BCAA to produce glutamate and BCKA, subsequently helping nucleotide synthesis, tricarboxylic acid cycle cycling, energy supply and redox homeostasis, that all benefit cancer cell survival and proliferation. Therefore, dissecting the molecular mechanism of BCAT2 regulation would provide a potential pharmaceutical target with regard to malignancies that are vulnerable to interference from BCAA metabolism. Interestingly, an elegant study revealed that BCAT2 was activated in the mRNA level by sterol regulatory element-binding protein 1 (SREBP1) in malic-enzyme-deleted PDAC cells [42]. On the other side, our group turned out post-translational modification of BCAT2 in PDAC cells [4,5]. Here, we identify that USP1 is the DUB for BCAT2 in response to BCAA availability. Functionally, increased BCAA upregulated the protein levels of BCAT2 and USP1 to promote PDAC cell growth *in vitro* as well as PanIN progression in KC mice.

USP1 is the most well-defined DUB and implicated in multiple aspects of DNA damage regulation including the Fanconi anemia pathway and translation process [43,44]. USP1 overexpression has been reported in many cancer types, like sarcoma and melanoma [45]. In this study, we identified upregulation of USP1 in ductal cells within PanIN and PDAC tissue. BCAA increases USP1 protein synthesis via inhibiting the GCN2-eIF2α pathway. In turn, elevated USP1 deubiquitylates BCAT2 at the K229 site to stabilize BCAT2 protein. At the early stage, this positive feedback enhances BCAA uptake and utilization and eventually induces the progression of PanIN cells to PDAC. ML323, acting as a highly specific inhibitor of USP1, decreased cell proliferation and clone formation in PDAC cells as well as pancreas weight in an orthotopic transplanted mice model, indicating that USP1 inhibition might be a potential therapeutic treatment for PDAC as it disrupts BCAT2-mediated BCAA catabolism.

PDAC is composed of a minority of tumor cells within a micro-environment containing an ECM, fibroblasts, immune cells and nerve cells [46]. A recent study demonstrates that BCAT1 is predominantly overexpressed in fibroblast cells to produce BCKA, which is excreted and taken up by PDAC cells to support protein synthesis and cell proliferation [3]. Meanwhile, another study found that BCKAs excreted from glioblastoma cells are taken up by macrophages to reduce its phagocytic activity [47]. Moreover, BCAA is required for the main proliferative status of Foxp3^+^ Treg cells in order to suppress the immune response [48]. However, the role of BCAA in the micro-environment of PDAC is not well known. Here we found that a higher BCAA diet enhanced ECM formation and macrophage cell recruitment. It would still be interesting to investigate the direct effect of BCAA in the micro-environment of PDAC at the early stage.

BCAT2 may potentiate acinar-to-ductal metaplasia (ADM), which is common in chronic pancreatitis and expedites the initiation of PanIN and facilitates adenocarcinoma [32]. Our data show that, physiologically, BCAT2 is exclusively expressed in acinar but undetectable in ductal epithelial cells. However, it is highly expressed in ductal epithelial cells from *Kras^G12D^*-driven PanIN lesions. It would be interesting to investigate the role of BCAT2 in acinar cells during the ADM process.

In summary, our study demonstrated that properly controlling the BCAA-BCAT2 metabolic axis through a BCAA-related dietary intervention (reduced BCAA content in the diet) and/or pharmaceutical inhibition of BCAT2 deubiquitylation, such as USP1 suppression, would provide rational therapy against PDAC.

## MATERIALS AND METHODS

For more details, see Supplementary Data.

## Supplementary Material

nwab212_Supplementary_files
